# Precipitation Modulates the Impact of Human Activities on Riverine Food Webs Revealed by Environmental DNA

**DOI:** 10.1002/ece3.73713

**Published:** 2026-06-01

**Authors:** Song Zhang, Xiaowei Zhang, Wenjun Zhong, Lewkowicz Anna, Yan Zhang, Loïc Pellissier

**Affiliations:** ^1^ State Key Laboratory of Pollution Control & Resource Reuse, School of the Environment Nanjing University Nanjing China; ^2^ Ecosystems and Landscape Evolution, Department of Environmental Systems Science ETH Zürich Zürich Switzerland; ^3^ Ecosystems and Landscape Evolution Swiss Federal Institute for Forest, Snow and Landscape Research (WSL) Birmensdorf Switzerland; ^4^ School of Ecology and Environmental Science Yunnan University Kunming China

**Keywords:** environmental DNA, food web, human activities, precipitation, river, spatial patterns

## Abstract

The functionality of global rivers has been adversely affected by environmental pollution and habitat degradation. We investigated the mechanisms through which climate variables modulate the impact of human activities on basin ecosystems. We compared the Han and Wei rivers, located on opposite sides of the Qinling Mountains, and modeled how climate modulates the effects of human activities on basin food webs. Water samples were collected from the Han and Wei Rivers, respectively, and we analyzed them using a combined environmental DNA (eDNA)‐hydrological coupling model and Empirical Dynamic Modeling. We found that the food web complexity showed a gradual decline from upstream to the outlet in the Han River, while the Wei River exhibited the opposite pattern. Food web complexity in the Han River was 67.18% higher than in the Wei River. The effects of human activities on ecological networks were associated with climate, with increased precipitation weakening the negative impact of human activities. Our results suggest that climate can modulate the effects of human activities on basin ecological networks. Additionally, basin ecosystems in arid regions demonstrate reduced resilience to withstand human activities under challenging climatic conditions.

## Graphical abstract

The effects of human activities on ecological networks are associated with climate, with increased precipitation weakening their negative impact.

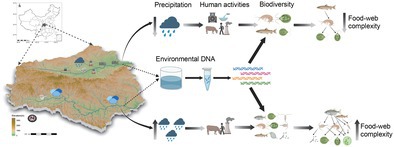

## 1 Introduction

Global river ecosystems are threatened by increasing human activities and climate change. The Kunming‐Montreal Global Biodiversity Framework (GBF) emphasizes the need to protect at least 30% of global river ecosystems by 2030, including restoring river biodiversity, improving water quality, and restoring the integrity and function of river ecosystems (Leadley et al. 2022). Nature‐positive depends on understanding how the services and processes provided by river ecosystems are influenced by human impacts and climate change. Empirical assessments have shown that increasing human pressure and climate change cause declines in the diversity of multi‐trophic organism groups in riverine systems (Moi et al. 2022). Multitrophic species declines, in turn, break down the relationship between biodiversity and ecosystem function, negatively affecting ecosystem functions like nutrient cycling and biomass production (Soliveres et al. 2016). As anthropogenic global changes intensify (Rocher‐Ros et al. 2023), how climate variables influence basin ecosystems' response to human activities remains unclear.

Human activities and climate change affect basin ecosystems and their functions through various mechanisms. Precipitation and human activities often co‐occur and could have joint effects on the food web structure (Blackman et al. 2022). Increased precipitation leads to a substantial influx of terrestrial agricultural pollutants and land‐derived dissolved organic matter into river ecosystems, supplying excess nutrients and contaminants (Canning and Death 2021). However, excessive nutrient load can lead to harmful algal blooms, increase light attenuation (Klug 2002), and decrease light availability in the water column, thus leading to a tipping point where primary production decreases. This underlying process may affect bacteria and phytoplankton production by shaping light penetration and nutrient availability, affecting the food web structure and overall river productivity (Merz et al. 2023; Binzer et al. 2016). Moreover, increased precipitation may lead to the spatial expansion of anthropogenic impacts. For instance, enhanced rainfall intensifies riverine runoff, promoting the downstream transport of substantial agricultural pollutants from upstream regions (Whitehead et al. 2009). This process exacerbates environmental pressures downstream and threatens both human water security and aquatic biodiversity at global scales (Vörösmarty et al. 2010). Concurrent water‐stress events under altered climate regimes have also been shown to reorganize river community structure and indicators across biogeographic regions (Espinar‐Herranz et al. 2025; Sabater et al. 2018). Consequently, variations in precipitation patterns may exacerbate the impacts of anthropogenic activities by facilitating increased influxes of pollutants and nutrients into riverine systems (Li et al. 2023). However, the interactive effect of precipitation and human activities on food web structure may thus diverge from empirical expectations (Bonnaffé et al. 2024). Therefore, assessing the complex impact of rainfall and anthropogenic influences within natural riverine environments is essential.

Mapping the spatial structure of food webs can reveal the mechanisms underlying the functionality and stability of basin ecosystems. Ecosystem functions are products of biological processes controlled by various trophic interactions (Thompson et al. 2012). For instance, trophic levels of the food web and the linkage between its trophic compartments shape ecosystem functioning (Lefcheck et al. 2015). Adopting a food web approach provides an opportunity to integrate specific interactions among multiple trophic levels to understand the functional consequences of human and climate disturbances on ecosystem functioning (Eisenhauer et al. 2019). Changes in human activities in basin ecosystems can affect the trajectory of network reorganization after disturbances, with the spatial distribution of these trajectories indirectly reflecting trends in basin ecosystem changes (Thompson et al. 2012). Therefore, mapping the spatial distribution of basin ecological networks aids in understanding how human impacts are spatially extended.

Environmental DNA (eDNA) metabarcoding can be used to recover species composition and, together with functional traits, infer food web structure. eDNA is the collection of DNA extracted from an environmental sample such as water, air, or sediment (Thomsen and Willerslev 2015). By collecting eDNA, we can screen samples for multiple taxonomic groups via metabarcoding (Deiner et al. 2016), creating a biodiversity assessment suitable for food‐web reconstruction. Some studies have shown that based on eDNA‐derived taxa occurrence data, a metaweb‐based approach can be used to examine local food‐web dynamics (Blackman et al. 2022; Li et al. 2023). In addition, eDNA co‐occurrence networks can successfully detect trophic interactions between marine communities, which could enhance our knowledge of spatio‐temporal variability of trophic interactions (Boyse et al. 2025). Thus, food webs built from environmental DNA metabarcoding data have arisen as a tool to explore interspecific interactions in ecological communities exposed to different human and environmental pressures.

Previous eDNA‐based food web studies have largely relied on discrete site‐level sampling without accounting for eDNA transport dynamics in river networks (Boyse et al. 2025; Blackman et al. 2022). Here, we advance this approach by coupling an eDNA‐hydrological model with Empirical Dynamic Modeling, enabling spatially continuous prediction of food web structure across entire river basins and assessment of how precipitation mediates human impacts on riverine food webs. This study aims to answer the following questions: (1) What are the spatial patterns of eDNA‐based food webs from upstream to the river outlet under different precipitation and human disturbances? (2) How do alterations in precipitation and human activities influence the structure of riverine food webs? (3) Is the impact of human activities on riverine food webs related to precipitation?

## 2 Method

### 2.1 Study Area

**FIGURE 1:**
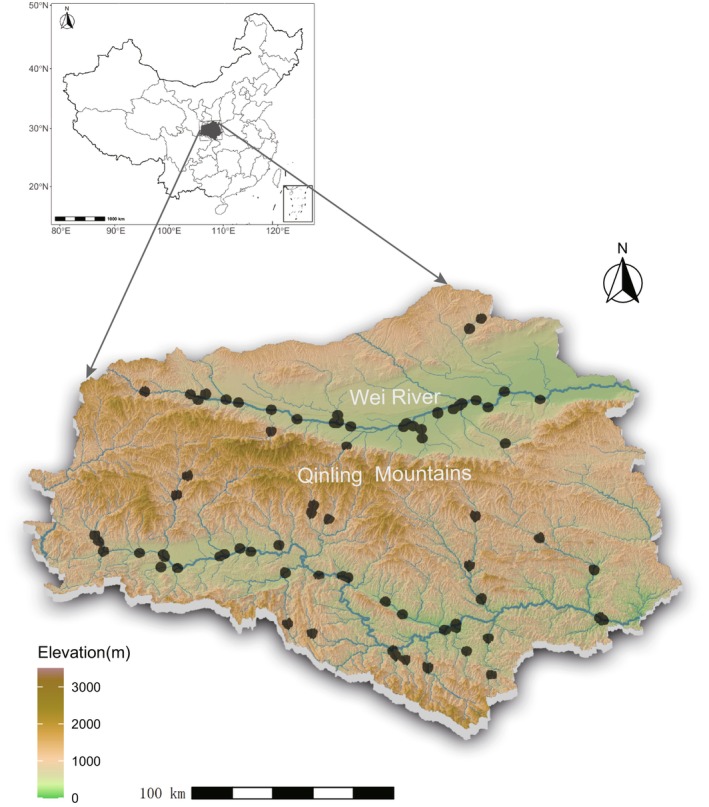
Sampling sites map in the Han River and the Wei River.

The Han River and the Wei River are situated on the southern and northern slopes of the Qinling Mountains, respectively. The Qinling Mountains serve as the watershed of the Yangtze River and the Yellow River. They are regarded as the natural boundary of geography and climate between northern and southern China (Hu et al. 2020). The Yangtze and Yellow River basins are China's largest and second‐largest rivers (Chen et al. 2020). The Yangtze River basin covers an area of 1.8 million km^2^, with a main channel length of 6300 km (Chen et al. 2020), while the Yellow River basin spans 752,400 km^2^ and has a main channel length of 5464 km. The Wei River and Han River basins receive an annual average precipitation of around 300 and 1000 mm, respectively (Tian et al. 2021; Wang et al. 2017). Beyond precipitation, the two basins differ substantially in geomorphology, land use, and nutrient inputs. The Wei River basin lies in a fault‐depression valley covered by loess plains under semi‐arid conditions, with a higher proportion of agricultural and urban land use (Wang et al. 2017; Liu et al. 2020). The Han River basin, on the southern slope of the Qinling Mountains, features gentler terrain with subtropical forested hills and greater natural vegetation cover (Ye 2016). Water quality studies indicate that the Wei River receives substantial nutrient loading from intensive dryland agriculture, while the Han River is primarily affected by precipitation‐driven transport of nitrogen and phosphorus into downstream reaches (Li et al. 2022; Tian et al. 2021; Kuo et al. 2019). These multi‐dimensional environmental contrasts provide a comprehensive comparative framework for examining how climatic, geomorphological, and anthropogenic factors jointly shape riverine food web structure. A total of 44 and 33 sampling sites were selected in the Han River and Wei River basins, respectively (Figure 1). The sampling locations include points along the tributaries and their downstream confluences to reflect the hierarchical structure of the dendritic river system. The nearest geographical distance between any two sampling sites was at least 30 km. This sampling density is consistent with recommendations for eDNA‐based river network biodiversity monitoring (Altermatt et al. 2023; Carraro et al. 2021). Simulation studies have shown that eDNA‐hydrological models achieve reliable prediction accuracy with ≥ 20 strategically placed sites, particularly when taxa are broadly distributed across the catchment (Carraro et al. 2021). Furthermore, the integrative nature of eDNA in flowing water—where each sample captures biological signals from upstream reaches (Deiner et al. 2016)—expands the effective spatial coverage beyond the immediate sampling locations.

### 2.2 Collection of Environmental DNA

Water samples were collected in the field between October and November 2022 following the previously established methods (Li et al. 2023; Altermatt et al. 2023). The samples were analyzed for multi‐biological groups, including fish, zooplankton, algae, and bacteria. Three replicates of 1 L of surface water were filtered per site through a 0.45 μm mixed cellulose membrane filter and then stored in individual sterile tubes at −20°C prior to DNA extraction (Li et al. 2023). Each replicate was extracted and analyzed independently. All equipment and utensils were cleaned with a 10% bleach solution before sample processing and between samples (Evans et al. 2017). At each site, 1 L of double‐distilled water (ddH_2_O) was also filtered as a blank control.

### 2.3 River Network Extraction and Climate and Footprint Mapping

The river network of the Han River and the Wei River was extracted through a TauDEM tool (Tarboton 1997) from a 90 m Digital Elevation Model (DEM) on the Google Earth Engine platform using the Han River's and Wei River's shapefiles generated from HydroBASINS (Lehner and Grill 2013). According to previous research (Lin et al. 2023), river discharge was estimated based on river width and channel slope. River widths were derived from Landsat 9 Collection 2 remote sensing imagery using the Google Earth Engine platform, employing the RivWidthCloud algorithm (Yang et al. 2020). Channel slope data were extracted from the basin's DEM. By applying Pitremove, D8Flowdir, and Aread8 methods, 1436 reaches for the Han River and 930 for the Wei River were obtained (Figure S1a,b), along with their respective hydrological information. Subsequently, the moveoutletstostreams algorithm was used to relocate each sampling point to its corresponding segment to avoid deviations in recorded latitude and longitude (Figure S2a,c). The ID of the river segment corresponding to each point was then identified. Furthermore, the outlet points of the Han River and Wei River basins were identified (Figure S2b,d).

To determine the average precipitation for the Han River and Wei River for 2022, we accessed the monthly average precipitation dataset for China from the National Tibetan Plateau Data Center. In brief, this data was generated based on the global 0.5° climate dataset published by CRU and the global high‐resolution climate dataset released by WorldClim (https://worldclim.org/), using the Delta spatial downscaling method. Finally, the average precipitation for the Han River and Wei River in 2022 was computed using the R packages “ncdf4” and “raster” (Pierce 2019; Hijmans et al. 2015). A 1 km buffer was created around each sample site, and the average values within the buffer were calculated using the mask function of the “terra” package (Hijmans et al. 2022).

Human pressure was quantified using the Human Footprint Index (HFP) (https://wcshumanfootprint.org/map/) (Venter et al. 2016a, 2016b). This index aggregates eight individual human pressures: (1) built environments, (2) crop lands, (3) pasture lands, (4) human population density, (5) nighttime lights, (6) railways, (7) roads, and (8) navigable waterways (Venter et al. 2016b). It ranges from 0 to 50 (the sum of all eight individual human pressures), with higher HFP values indicating greater human pressure (Venter et al. 2016a). In addition, to measure the anthropogenic stress on the Han River and Wei River, the global human footprint value in 2018 (Mu et al. 2022) was used as a metric by creating a 1 km buffer around each sample site and calculating the average values within the buffer using the mask function of the terra package.

### 2.4 DNA Extraction, PCR Amplification, and Bioinformatics

All membrane discs (including blank controls) were processed using a DNeasy Blood Tissue Kit (QIAGEN, Germany). Polymerase chain reaction (PCR) assays were conducted with three primer sets. Specifically, a universal primer pair (Teleo2_F: AAACTCGTGCCAGCCAC and Tele02_R: GGGTATCTAATCCCAGTTTG) was used to amplify the fragment of the 12S rRNA genes to detect fish (Taberlet et al. 2018), a universal eukaryotic primer pair (1389F: TCCCTGCCHTTTGTACACAC and 1510R: CCTTCYGCAGGTTCACCTAC) was used to amplify the fragment of the V9 region of 18S rRNA genes to detect zooplankton and algae (Amaral‐Zettler et al. 2009), and the fragment of the V3 region of 16S rRNA genes was amplified using the primer pair (341F: ACCTACGGGRSGCWGCAG and 518R: ATTACCGCGGCTGCTGG) to detect bacteria (Klindworth et al. 2013). Purified PCR products were pooled in a single tube with equal volume quantities for library preparation and sequencing. Depending on the PCR amplicon size, sequencing was conducted on the Illumina MiSeq PE150 or PE 300 platform (Illumina, USA).

Forward and reverse sequences were merged using the “–fastq_mergepairs” script in the VSEARCH pipeline (Rognes et al. 2016). Subsequently, the barcode_splitter script was used to split the data by sample ID. Demultiplexing and primer trimming were performed on all sequences assembled by VSEARCH in each sample using Cutadapt software (Martin 2011), with a maximum allowed mismatch error rate of 0.1. All sequences were clustered as amplicon sequence variants (ASVs) through SWARM (Mahé et al. 2014), using a minimum distance of one nucleotide between each sequence (*d* = 1). The “‐‐uchime_denovo” command in VSEARCH was performed to check and remove chimeras. All ASVs were assigned by a lowest standard ancestor algorithm, ecotag from OBITOOLs (Boyer et al. 2016) with a fish barcode reference database, which was built using all sequences and taxonomic information downloaded from NCBI. Taxonomic annotation of all ASVs in bacteria was assigned against the SILVA database v138 (Quast et al. 2012); all ASVs in eukaryotic algae and zooplankton against the PR2 database v4.14 (Guillou et al. 2013) and a custom reference database (NCBI Genbank database downloaded in March 2022 and an indigenous database).

### 2.5 Inference of the Aquatic Food Web Based on eDNA

#### 2.5.1 Food Web eDNA Data

We assembled 44 and 33 environmental DNA (eDNA) datasets from the Han and Wei Rivers, respectively. Each dataset includes eDNA abundance data for multiple taxonomic groups. Datasets were selected based on the following criteria: (1) the number of trophic levels is at least 2; (2) taxa are identified to the species level or the best possible taxonomic level (typically species level); (3) to exclude low‐frequency species with too many zero values in their samples, we retained only those detectable species at more than half of the sampling sites. This occurrence threshold was applied as a data quality filter to exclude species with sporadic detections likely resulting from false positives or stochastic eDNA signals, a well‐recognized issue in eDNA metabarcoding (Evans et al. 2017; Ficetola et al. 2015; García‐Machado et al. 2023). This criterion balances the need to minimize noise from unreliable detections while retaining ecologically meaningful species for food web construction. Species excluded by this filter were primarily those with low detection frequency, which typically occupy peripheral positions in food webs and contribute minimally to core structural metrics such as connectance and link density (Blüthgen et al. 2008; Dunne et al. 2002). As a result, 77 and 63 species were retained for the Han and Wei Rivers (Tables S1 and S2), respectively. In the Han and Wei River basins, we retained 30 and 26 fish species, including large invertebrate predators, omnivores, large herbivores, small herbivores, and mixotrophs. We retained 9 and 6 protozoa species in the Han and Wei River basins, respectively, as well as 13 and 8 metazoan, 14 eukaryotic algae, and 11 and 9 bacteria. Before the Empirical Dynamic Modeling analysis, all abundance data were normalized.

#### 2.5.2 Inferring Interactions Among Species

For the Han River and Wei River eDNA datasets, we mapped the potential trophic interaction links across all potential species pairs in each dataset using convergent cross mapping (Sugihara et al. 2012; Merz et al. 2023; Zhao et al. 2023) (CCM). CCM is a nonlinear association test that estimates the extent to which changes in one variable correlate with changes in another by measuring cross‐prediction (Merz et al. 2023). CCM is based on Takens's theorem, which proves that it is possible to construct a shadow version of the original attractor of a dynamical system by substituting time lags of the observable variables for the unknown variables (Sugihara et al. 2012; Deyle and Sugihara 2011). In our study, the variables are the species relative abundances, where their association, inferred by CCM, represents a trophic link. For example, consider species 1 (phytoplankton) and species 2 (herbivores). We assess whether the abundance of species 1 is significantly associated with the abundance of species 2 across sites. With the CCM method, the non‐linear correlations of reads of species 1 with species 2 allow for the estimation of dependencies between pairs of species, a process known as cross‐mapping between variables (Sugihara et al. 2012). The stronger the signature of the estimated association, the better the cross‐map estimate. We used the R package rEDM and utilized species abundance matrices across multiple trophic levels as input data to infer the directionality and strength of interspecific interactions. Following Zhao et al. (2023), we used the direction from species to species to represent either the predation or the being‐predated relationship between two different trophic levels. Based on the CCM method, we obtain information on trophic interaction linkages among species using the abundance matrix of multi‐trophic level species. As the eDNA abundance data used are located at different spatial scales, we rescale them using the function scale in the R package base (v.4.1.0).

### 2.6 Quantifying Food Web Structure and Characteristics

Once trophic interactions among species have been inferred, the food‐web structure can be constructed based on predation or competition interactions. We can calculate food‐web characteristics by integrating species causal‐interaction data with their site‐specific eDNA occurrence records. We used the following indices to identify food web metrics. The measurement of interactions, known as connectivity (L), has been used as the common description of food web complexity (Havens 1992; Dunne et al. 2002). Connectivity is simply the total number of interactions (L) within a network (Okuyama and Holland 2008). In addition, linkage density is another widely used measure of food web complexity, calculated as the average number of links per species, or the connectivity divided by species richness (L/S) (Havens 1992). Omnivory is defined as having a mixed trophic‐level diet. The level of omnivory has been proposed as a measure of food‐web stability, and weak omnivorous interactions are likely to lead to a more stable food web (Wootton 2017). Trophic level and level of omnivory of species were calculated using the methods stated in Williams and Martinez (2004), and omnivory was averaged over all consumers in the food web as a community‐level index. In addition, we applied generalized linear models (GLM) to explore the spatial patterns of food web metrics from upstream to outlet.

### 2.7 Constructing a High‐Resolution Spatial Distribution Prediction Model for Food Web

The environmental DNA‐hydrological model was implemented using the previously proposed methods (Carraro et al. 2020; Carraro and Altermatt 2024). The spatial distribution patterns of the riverine ecological network were established using the environmental DNA‐hydrological coupling model, following previously validated methods (Carraro et al. 2020; Carraro and Altermatt 2024). In brief, following the principle that “the decay of organic matter in straight rivers under steady water flow conforms to first‐order degradation kinetics,” a model of eDNA concentration degradation dynamics was constructed at specific river nodes upstream of the catchment area. The decay rate constant (*τ*) governs the exponential decline of eDNA concentration with travel time downstream. Empirical estimates of eDNA decay rates under relevant environmental conditions were used to parameterize the model (Shogren et al. 2017). Although eDNA decay rates vary among taxa, first‐order kinetics has been shown to apply broadly across diverse organisms, with decay rates driven primarily by environmental conditions rather than taxonomic origin (Shogren et al. 2017). We applied this framework for relative spatial comparisons of eDNA‐derived biodiversity patterns rather than absolute quantification of single‐taxon concentrations. A riverine environmental DNA sequence dynamics model was derived by combining the ratio of estimated eDNA sequence numbers to eDNA concentration estimates. Using algorithms such as pit filling, flow direction analysis, flow statistics, and catchment area modeling, river networks were extracted from the digital elevation model, and sub‐watershed differentiation of rivers was conducted. eDNA transport through the river network was modeled as an advective process, where eDNA particles move downstream with the water flow. River discharge at each network node was estimated from remotely sensed river widths and channel slopes (Lin et al. 2023), following hydrological scaling laws. Flow velocity was derived from discharge and channel cross‐sectional area. The model accounts for dilution effects at confluences, where upstream tributaries merge. Relevant hydrogeomorphic covariates (e.g., water depth, river width, flow rate, topography, and geomorphology) influencing the environmental DNA dynamics model were incorporated to construct a coupled watershed biodiversity distribution model integrating eDNA with hydrology and geology. The model was calibrated using observed eDNA data at the 44 (Han River) and 33 (Wei River) sampling sites, optimizing the decay rate parameter and eDNA production rates to minimize the discrepancy between predicted and observed eDNA concentrations. To address detection probability, three replicate samples were collected at each site, and only species detectable at more than half of the sampling sites were retained in the analysis. Uncertainties in flow rate estimates were propagated through the model by using the variance of discharge estimates derived from the RivWidthCloud algorithm (Yang et al. 2020). The model's predictive performance was validated by leave‐one‐out cross‐validation, comparing predicted and observed ecological network characteristics at held‐out sampling sites.

After analyzing the species composition and trophic network interactions at different sites, the model used environmental DNA monitoring data and hydrogeomorphic measurements from different sites as input variables. The model was then applied to obtain the ecological network characteristic parameters (e.g., connectivity, linkage, etc.) of different differentiated river segments, and ultimately, spatial patterns of the watershed ecological network were drawn based on river connectivity.

### 2.8 Quantifying the Effects of Human Activity on the Food Web

The effects of human activities and precipitation on food web characteristics were assessed using PLS‐PM, which is suited for modeling complex causal pathways with multiple latent variables under small sample sizes (Tenenhaus et al. 2005). PLS‐PM is robust to multicollinearity; all VIF values were confirmed to be below five (Sanchez 2013). The model included observed variables such as elevation, mean annual temperature, mean annual precipitation, human footprint, fish richness, zooplankton richness, eukaryotic algae richness, number of links, and link density. Eight latent variables were specified: hydrological factors (discharge), climatic factors (mean annual precipitation), human activity factor (human footprint), fish diversity (fish richness), zooplankton diversity (zooplankton richness), algal diversity (eukaryotic algae richness), and food web complexity (number of links, link density). To ensure optimal model interpretability, observed variables with loadings less than 0.7 were excluded, based on the relationships between observed and latent variables in the external model. Ultimately, we modeled the adjusted latent variables, achieving a goodness‐of‐fit (GOF) of 0.71. In addition, individual relationships between environmental factors and food web metrics were additionally assessed using Spearman correlations and generalized linear models.

## 3 Result

### 3.1 Aquatic Biodiversity in the Han and Wei River

**FIGURE 2:**
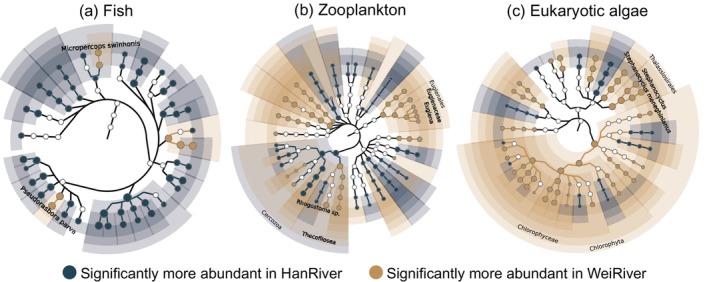
Differences in the lineages of fish (a), zooplankton (b), and eukaryotic algae (c) between the Han River and Wei River. The size of the circles is proportional to the logarithm of the absolute difference in relative abundance between the Han River and Wei River biological communities.

The richness at the species level of fish, zooplankton, and eukaryotic algal communities in the Han River was significantly higher than in the Wei River (Figure S4a–c), and significant differences in community structure were also observed (Figure S4d–f). Additionally, GraPhlAn (Asnicar et al. 2015) was utilized to illustrate the differential taxa across various taxonomic levels, from phylum to species (Figure 2). The abundance of 14 species in the Han River was significantly higher for fish than in the Wei River. In contrast, the abundance of *Pseudorasbora parva* and *Micropercops swinhonis* exhibited the opposite trend (Figure 2a). For zooplankton, the abundance of the Cercozoa phylum in the Han River was significantly higher than in the Wei River. At the same time, the Thecofilosea class, Euglenales order, Euglenaceae family, Euglena genus, and *Rhogostoma* sp. species were the opposite (Figure 2b). For eukaryotic algae, most taxa exhibiting significant differences showed lower abundances in the Han River compared to the Wei River, including the Chlorophyta phylum, Chlorophyceae class, Thalassiosirales order, *Stephanocyclus* genus, and *Stephanocyclus meneghinianus* species (Figure 2c). Additionally, the Han River and Wei River exhibited more distinct taxa in zooplankton and eukaryotic algal lineages than in fish lineages.

### 3.2 Food Web Structure

**FIGURE 3:**
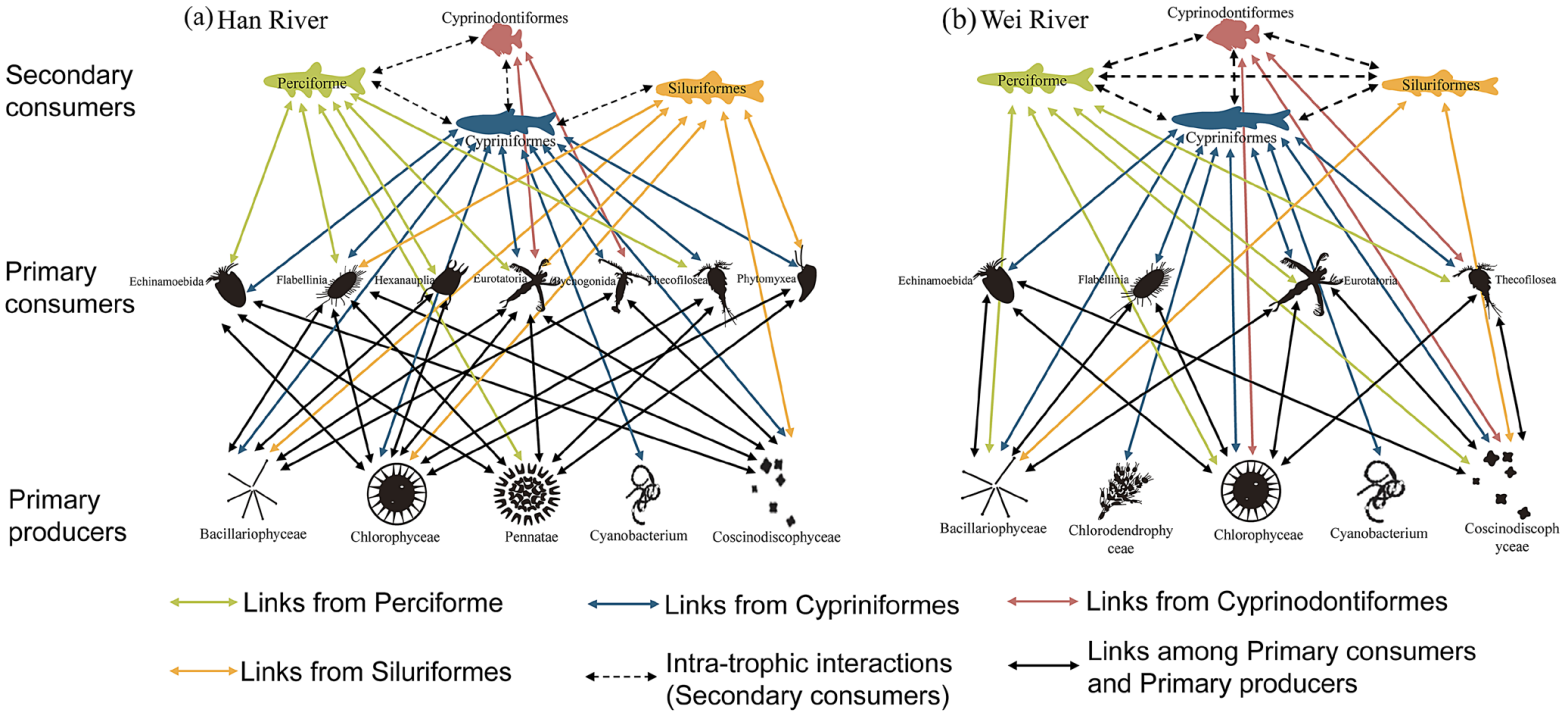
Food web structure of the Han River (a) and Wei River (b) inferred from eDNA detection. Taxa are arranged by trophic level: Primary producers (bottom), primary consumers (middle), and secondary consumers (top). Colored arrows indicate trophic links originating from secondary consumers: Green = links from Perciforme, blue = links from Cypriniformes, orange = links from Siluriformes, red = links from Cyprinodontiformes. Black solid arrows represent links among primary consumers and primary producers. Black dashed arrows indicate intra‐trophic interactions among secondary consumers.

At the species level, the food web in the Han River included 30 fish species, 22 zooplankton species, 14 algal species, and 11 bacterial species, totaling 77 nodes and 1146 links (Table S1). In contrast, the food web in the Wei River included 26 fish species, 14 zooplankton species, 14 algal species, and nine bacterial species, totaling 63 nodes and 524 links (Table S2). To enhance visualization, we depicted the food web structures of the Han River and Wei River at the high trophic level by order level and at the mid and low trophic levels by class level (Figure 3). We noted that both the Han River and Wei River at the high trophic level included four common orders: Perciformes, Cyprinodontiformes, Siluriformes, and Cypriniformes. Notably, the high‐trophic Siluriformes possessed six trophic links to mid and low trophic levels in the Han River, whereas the Wei River had only two. The Cypriniformes order represented the highest number of trophic links in both rivers, but the predation relationships varied. The Han River and Wei River contained seven and four classes at the mid trophic level, respectively. Both rivers shared four classes (Echinamoebida, Flabellinia, Eurotatoria, Thecofilosea); nevertheless, Hexanauplia, Pycnogonida, and Phytomyxea were exclusive to the Han River's food web. At the low trophic level, four classes were shared by both rivers: Coscinodiscophyceae, Cyanobacterium, Chlorophyceae, Bacillariophyceae. However, Pennatae was exclusive to the Han River's food web, and Chlorodendrophyceae was exclusively present in the food web of the Wei River (Figure 3).

### 3.3 The Spatial Patterns of Riverine Ecological Networks

**FIGURE 4:**
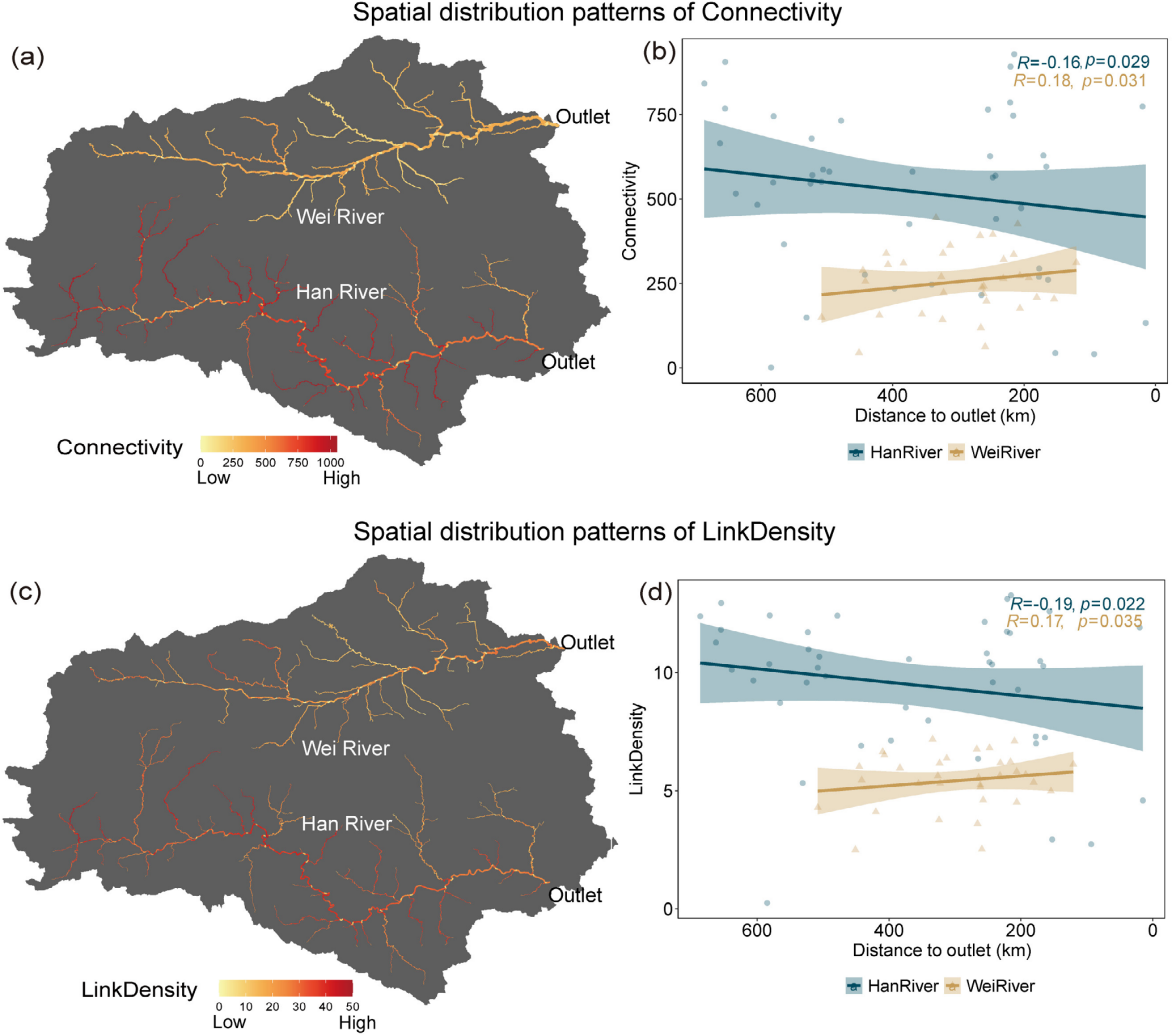
Spatial distribution patterns of food web connectivity (a, b) and link density (c, d). Panels (a) and (c) show model‐predicted values across the river network, with color gradients ranging from low (yellow) to high (red) values. Panels (b) and (d) show the relationship between distance to outlet (km) and true observed values at eDNA sampling sites. Blue circles and fitted lines represent the Han River; tan triangles and fitted lines represent the Wei River. Shaded bands indicate 95% confidence intervals. Pearson correlation coefficients and *p*‐values are shown for each river.

The model results show that distinct spatial variations in food web complexity (connectivity and link density) were observed across the upstream, midstream, and downstream sections of both the Han River and Wei River (Figure 4a,c). Similarly, food‐web complexity inferred from true eDNA sampling sites exhibited significant variation from the upstream to the outlet. For instance, the ecological complexity showed a gradual decline from upstream to the outlet in the Han River (Figure 4b,d), while the Wei River exhibited the opposite pattern.

### 3.4 The Effect of Human Activities on Food Web Metrics Under Different Climatic Conditions

**FIGURE 5:**
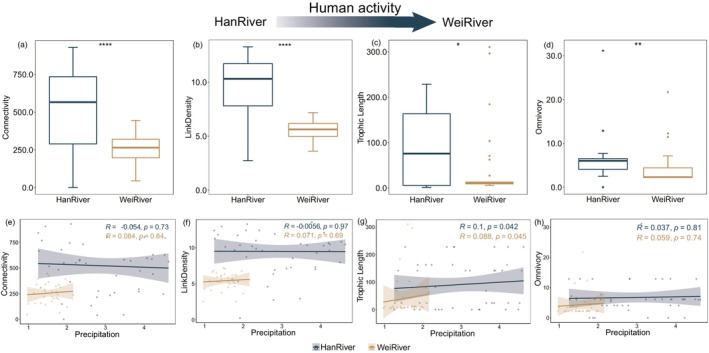
Changes in Number of Links (a), Link Density (b), Trophic Length (c), and Omnivory (d) under differential anthropogenic pressures. The relationships between precipitation and the Number of Links (e), Link Density (f), Trophic Length (g), and Omnivory (h). *p < 0.05, **p < 0.01, ****p < 0.0001.

Under intensified human impacts, river food webs showed significant reductions in several links, link density, trophic length, and omnivory levels (*p* < 0.05; Figure 5a–d).

However, food web structure exhibited no significant correlation with precipitation, except for trophic length (Figure 5e–h). Trophic length showed a weak positive association with precipitation.

### 3.5 Mechanisms by Which Human Activities Affect River Food Webs

**FIGURE 6:**
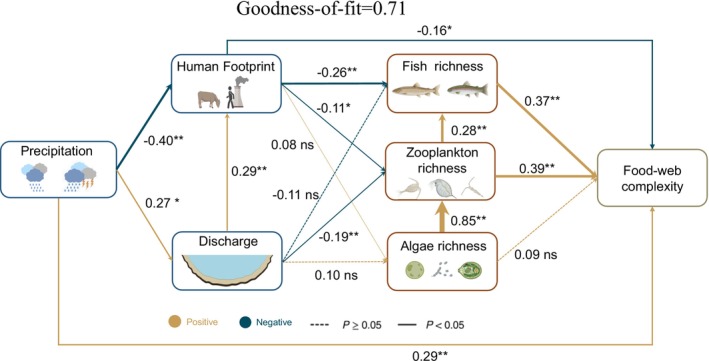
Mechanistic insights into anthropogenic impacts on the riverine food web. The PLS‐PM model illustrated the direct and indirect effects of human activities and precipitation on biodiversity and the complexity of the ecological network. The model fits well with the data (GOF = 0.71). Solid lines indicate significant effects, while dashed lines represent nonsignificant effects. The numbers on the arrows represent standardized coefficients, reflecting the intensity of influence one factor exerts on another. The width of the arrows is weighted according to the standardized coefficients. **p < 0.01, *p < 0.05, ns: *p* > 0.05.

Human activities influence river food webs primarily through two pathways. Firstly, precipitation directly decreased human activity density (Direct effect = −0.40; *p* < 0.01; Figure 6), which caused an increase in fish richness, leading to increased biodiversity at higher trophic levels, thereby enhancing food web complexity. Secondly, the increase in river discharge directly increased the intensity of human activity disturbances (Direct effect = 0.29; *p* < 0.01; Figure 6), which affected food web complexity by altering biodiversity. Overall, the impacts of human activities on the food web were modulated by precipitation.

## 4 Discussion

This study revealed the mechanisms by which precipitation mediated the impacts of human activities on watershed ecosystems through spatial variations in food webs constructed using environmental DNA. We found that the spatial distribution patterns of food webs differed between river basins. Under intensified anthropogenic pressures, river food webs exhibited significant reductions in several links, link density, trophic length, and omnivory levels. The influence of human activities on food web structure is primarily mediated through their negative impacts on the richness of multi‐trophic communities, thereby indirectly impacting food web complexity. The intensity of human activities is regulated both directly and indirectly by precipitation.

In the Wei River, where human activity is intense and precipitation is low, food web complexity increases from upstream to the outlet. At the same time, the trend is reversed in the Han River. Multiple mechanisms could explain the phenomenon. We observed that discharge in the Wei River increases upstream to the outlet (Figure S3c) and positively correlates with food web complexity (Figure S5a,b). As river discharge increases, it transports greater quantities of upstream‐derived nutrients downstream, thereby enhancing resource availability for multiple taxa (Wang et al. 2017). This process ultimately intensifies predator–prey interactions and competitive relationships in downstream reaches.

In contrast, in the Han River, discharge negatively correlates with food web complexity (Figure S5a,b). Due to the higher precipitation in the Han River (Figure S3d), there is an increase in terrestrial pollutants entering the river. Published water quality monitoring data from the Han River basin have demonstrated that water quality deteriorates from upstream to downstream, driven primarily by the transport of nitrogen and phosphorus from agricultural non‐point sources and domestic sewage during precipitation events (Li et al. 2022; Tian et al. 2021; Kuo et al. 2019). The simultaneous increase in discharge and nutrient loading causes these pollutants to accumulate downstream, leading to eutrophication and water quality degradation (Li et al. 2022). Elevated nutrient concentrations, particularly nitrogen and phosphorus, have been shown to weaken food web complexity by promoting eutrophication and disrupting trophic interactions (Binzer et al. 2016). These findings indicate that human activities and precipitation may influence the spatial distribution of river food web characteristics by mediating discharge.

When human activities intensified, the number of links, link density, trophic length, and omnivory levels of river food webs decreased significantly. Previous studies indicated that watersheds subject to intense human activity exhibited simplified food webs (Bonnaffé et al. 2024). These findings were consistent with the observations in this study. For centuries, human activities have caused profound changes in fish communities through species loss and declining abundance in fish trophic guilds (Lefcheck et al. 2021). The decline of high trophic‐level fish directly leads to the gradual disappearance of apex and mid‐level predators from aquatic ecosystems (Estes et al. 2011). The loss of apex predators releases lower trophic levels from top‐down control, leading to blooms of lower‐level organisms and a compressed number of trophic transfers (Daskalov et al. 2007). These cascades effectively remove one or more “rungs” from the trophic ladder, shortening maximum chain length by one or more steps (Thompson et al. 2012). In addition, changes to the spatial arrangement and composition of habitat can reduce the number and alter the configuration of trophic interactions within species networks (Bartley et al. 2019). Land use is the main factor in habitat diversity change. In landscapes characterized by high physical heterogeneity, food webs tend to exhibit diverse resource bases, elevated omnivory, and a high proportion of trophic interactions (Rooney et al. 2008). These patterns align with the multitrophic biodiversity–ecosystem functioning (BEF) framework, which holds that biodiversity across trophic levels is essential for ecosystem multifunctionality (Soliveres et al. 2016; Lefcheck et al. 2015; Eisenhauer et al. 2019), and with recent riverine evidence that multitrophic diversity loss weakens food web functioning. Moreover, food web theory predicts that higher connectance and weak omnivorous interactions confer stability and robustness against perturbation (Dunne et al. 2002; McCann et al. 1998). Our results—showing significant reductions in these stability‐related metrics under anthropogenic pressure—are consistent with findings that environmental stress drives food‐web destabilization through biodiversity loss (Bonnaffé et al. 2024; Canning and Death 2021). Our study extends this literature by demonstrating that precipitation regime modulates the intensity of these destabilizing effects, highlighting a climatic dimension largely unexplored in prior research on riverine food‐web stability.

The effects of human activities on ecological networks were associated with climate. Our results suggest that increased precipitation may weaken the negative impact of human activities on food web complexity through several interconnected mechanisms. First, higher discharge dilutes anthropogenic pollutant concentrations in the water column, reducing chemical stress on aquatic biota (Vörösmarty et al. 2010; Whitehead et al. 2009). This mechanism is supported by extensive evidence that low‐flow conditions concentrate pollutants and degrade water quality, leading to the loss of pollution‐sensitive taxa that serve as key nodes in aquatic food webs (Dewson et al. 2007; Sabater et al. 2018; Stubbington et al. 2017). Second, sufficient precipitation maintains hydrological connectivity and habitat heterogeneity, which are critical for sustaining complex trophic interactions (Tockner et al. 2000; Rooney et al. 2008). Habitat fragmentation under reduced flows can isolate populations and erode species interactions (Datry et al. 2014; Leigh et al. 2016), and the combination of water stress and pollution can have synergistic negative effects on aquatic communities (Stubbington et al. 2019). Third, precipitation‐driven hydrological pulses provide terrestrial nutrient subsidies that broaden the trophic base, potentially counteracting the simplifying effects of anthropogenic disturbance (Jardine et al. 2012; Junk et al. 1989). While our results suggest a modulating role of precipitation, direct measurements of pollutant concentrations and habitat connectivity are needed to confirm the proposed mechanisms. Future studies incorporating concurrent measurements of pollutant concentrations, habitat conditions, and biological communities along precipitation gradients would help validate these proposed pathways.

Our sampling was conducted during a single period (October–November 2022), which may not fully capture seasonal dynamics in species composition and food‐web structure. This single‐period design may influence network interpretation, as seasonally absent taxa (e.g., migratory species or seasonally reproducing invertebrates) would not be captured, potentially affecting network size and metrics such as connectance and link density (Blackman et al. 2022). Additionally, trophic interactions can shift seasonally with changes in prey availability and ontogenetic diet shifts (Winemiller 1990), suggesting that the food‐web topology presented here represents a subset of the full annual interaction network. However, because both rivers were sampled during the same period, this temporal bias is consistent across systems, preserving the validity of between‐river comparisons. Future multi‐seasonal sampling would help assess the temporal stability of these patterns. The convergent cross mapping (CCM) method used to infer species interactions requires careful interpretation. CCM differs from conventional correlations by testing for causal coupling based on Takens's theorem, wherein the historical states of one variable contain recoverable information about another (Sugihara et al. 2012). However, CCM detects nonlinear dynamic dependencies rather than directly observing trophic interactions, and detected associations may also reflect shared environmental responses, indirect effects, or non‐trophic biotic coupling (Chang et al. 2017). To mitigate misinterpretation, we restricted inference to cross‐trophic‐level species pairs and assigned interaction directionality following Zhao et al. (2023). Nevertheless, eDNA‐derived abundances reflect co‐occurrence patterns rather than direct dietary evidence (Hansen et al. 2018), and the inferred links should therefore be interpreted as statistically supported potential trophic interactions requiring future validation through complementary approaches such as dietary metabarcoding or stable isotope analysis. Finally, the > 50% site occurrence threshold may exclude naturally rare specialist taxa, but sporadically detected species are difficult to distinguish from false positives in eDNA metabarcoding (Ficetola et al. 2016; Guillera‐Arroita et al. 2017), and their removal has minimal effects on core food‐web metrics (Dunne et al. 2002). Despite these limitations, our dataset spans a broad range of trophic levels from primary producers to strict piscivores, enabling meaningful food‐web comparisons.

## 5 Conclusions

This study reveals how precipitation regulates the impact of human activities on riverine ecosystems using environmental DNA. Our main findings are as follows: (1) Watersheds with higher annual precipitation and less human disturbance exhibited higher network metrics; (2) the spatial pattern of food web complexity showed an inverse trend from upstream to outlet between river basins; (3) the effects of human activities on ecological networks were modulated by precipitation. These data highlight that food web complexity in arid watershed ecosystems is reduced under the combined pressures of intensified human activity and low precipitation, indicating greater vulnerability of these ecosystems to ongoing anthropogenic disturbance. Our findings imply that in arid regions, reducing anthropogenic pressures during low‐precipitation periods may be critical for maintaining food web complexity and ecosystem stability.

## Author Contributions

**Song Zhang:** conceptualization (lead), data curation (lead), formal analysis (lead), investigation (lead), methodology (lead), project administration (lead), resources (lead), software (lead), validation (lead), visualization (lead), writing – original draft (lead), writing – review and editing (lead). **Xiaowei Zhang:** conceptualization (equal), data curation (equal), funding acquisition (lead), project administration (equal), resources (equal), supervision (lead), writing – original draft (equal), writing – review and editing (equal). **Wenjun Zhong:** formal analysis (equal), methodology (equal), software (equal). **Lewkowicz Anna:** writing – review and editing (equal). **Yan Zhang:** writing – review and editing (supporting). **Loïc Pellissier:** conceptualization (equal), supervision (equal), writing – review and editing (equal).

## Conflicts of Interest

The authors declare no conflicts of interest.

## Supporting information


**Table S1:** ece373713‐sup‐0001‐AppendixTable1.xlsx.

**Table S2:** ece373713‐sup‐0002‐AppendixTable2.xlsx.

**Figure S1:** Map of reach in the Han (a) and Wei (b) River basins.
**Figure S2:** The sampling sites in the Han River and Wei River basins correspond to the reach (a, c) and basin outlet locations (b, d), respectively.
**Figure S3:** The spatial distribution patterns and differences of average precipitation (a, b, c), human footprint (d, e, f) in the Han and Wei River. The spatial distribution patterns represent the average values of average annual precipitation and human footprint within a 1 km buffer zone surrounding each samplesite.
**Figure S4:** The alpha diversity and beta diversity of fish (a, d), zooplankton (b, e), and eukaryotic algae (c, f) communities between the Han and Wei River.
**Figure S5:** Relationship between food web complexity and flow in the Han and Wei River basins (a, b).
**Table S1:** Species names from different trophic levels used in the food web analysis of the Han River Basin.
**Table S2:** Species names from different trophic levels used in the food web analysis of the Wei River Basin.

## Data Availability

Analysis code and data can be found on Zenodo (https://zenodo‐rdm.web.cern.ch/records/15705835).
