# Supplementary material for: Precipitation Modulates the Impact of Human Activities on Riverine Food Webs Revealed by Environmental DNA
Source: Ecol Evol. 2026 Jun 1;16(6):e73713. doi: 10.1002/ece3.73713 (PMC13238525; doi:10.1002/ece3.73713)
Supplement: Supplementary file 3 — Figure S1: Map of reach in the Han (a) and Wei (b) River basins. Figure S2: The sampling sites in the Han River and Wei River basins correspond to the reach (a, c) and basin outlet locations (b, d), respectively. Figure S3: The spatial distribution patterns and differences of average precipitation (a, b, c), human footprint (d, e, f) in the Han and Wei River. The spatial distribution patterns represent the average values of average annual precipitation and human footprint within a 1 km buffer zone surrounding each samplesite. Figure S4: The alpha diversity and beta diversity of fish (a, d), zooplankton (b, e), and eukaryotic algae (c, f) communities between the Han and Wei River. Figure S5: Relationship between food web complexity and flow in the Han and Wei River basins (a, b). Table S1: Species names from different trophic levels used in the food web analysis of the Han River Basin. Table S2: Species names from different trophic levels used in the food web analysis of the Wei River Basin. [file ECE3-16-e73713-s002.docx]

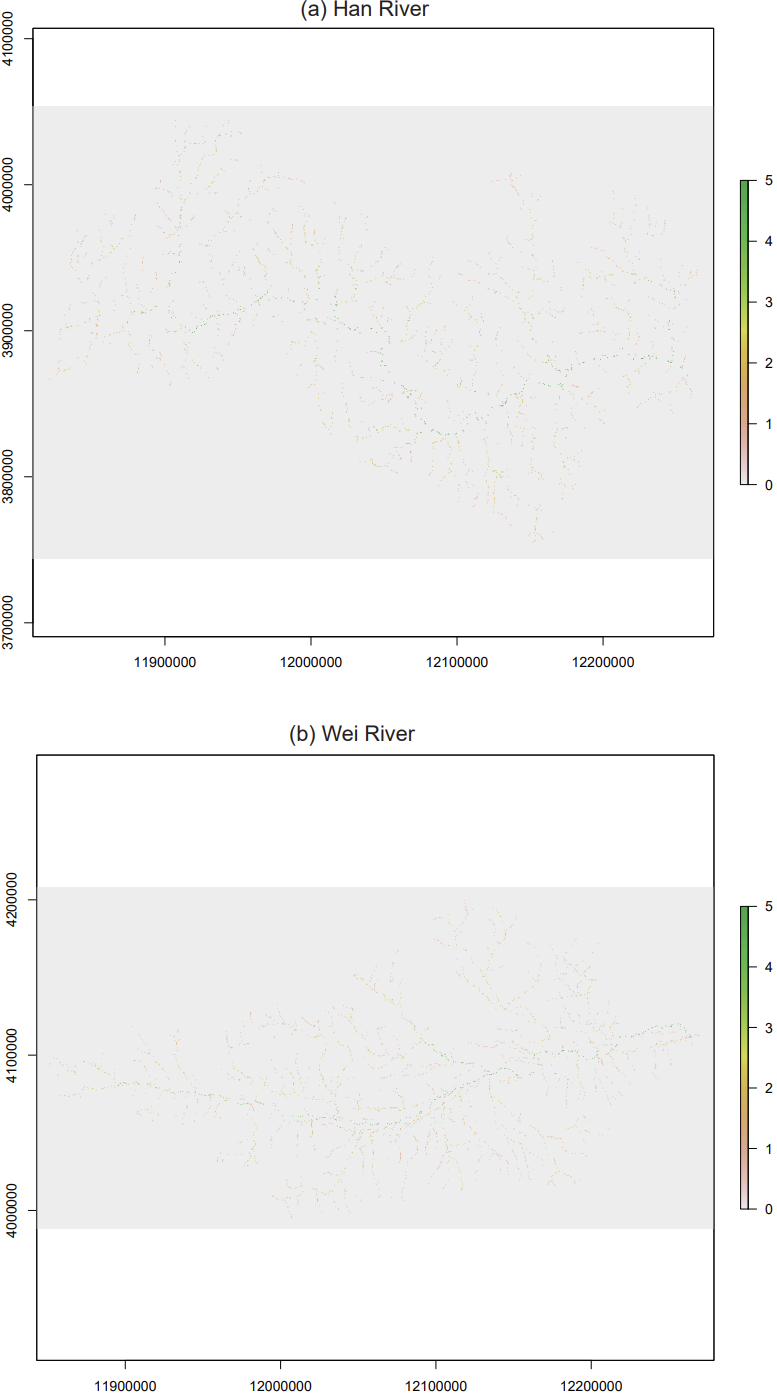
**Figure S1.** Map of reach in the Han (a) and Wei (b) River basins.


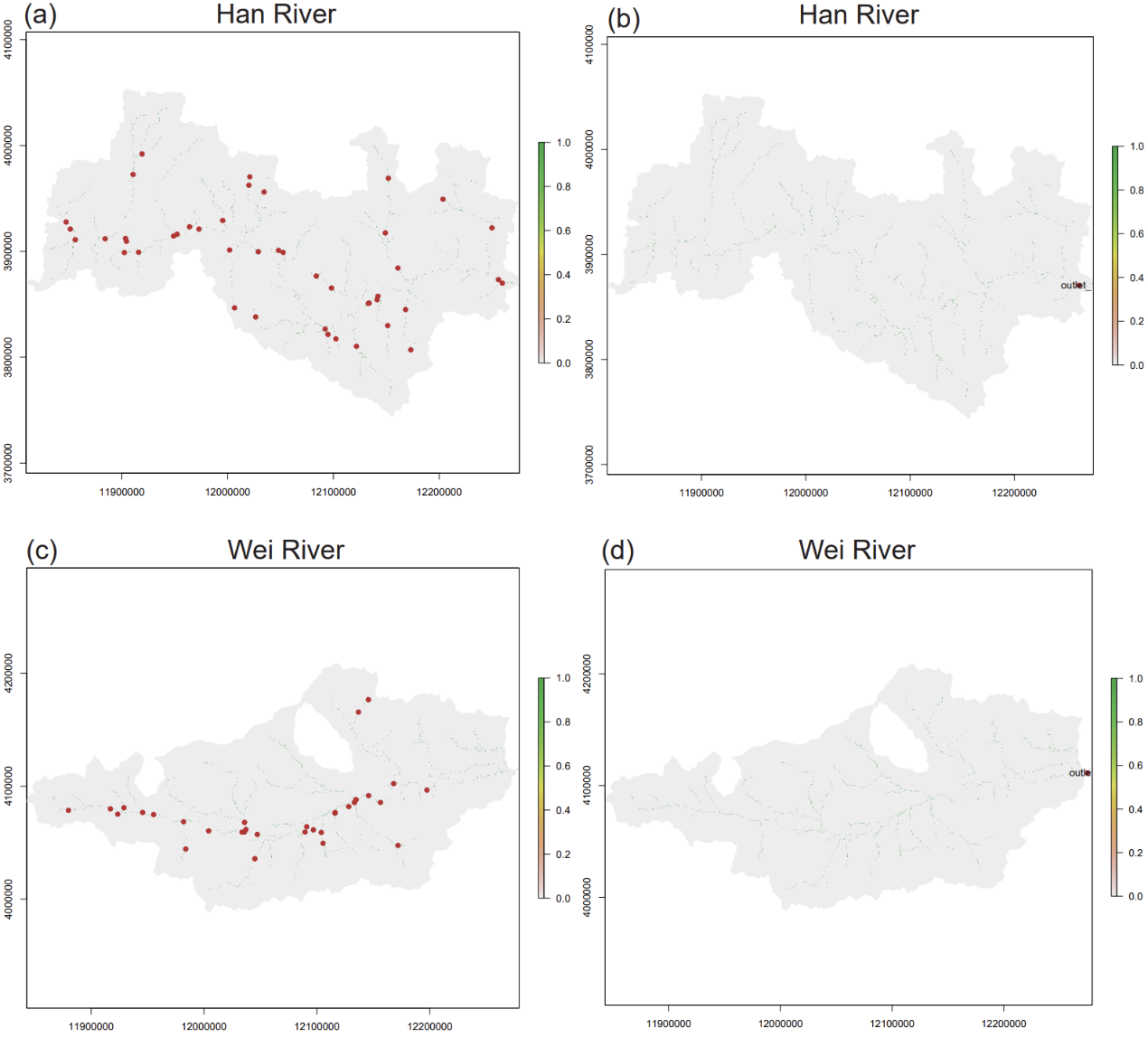
**Figure S2.** The sampling sites in the Han River and Wei River basins correspond to the reach (a, c) and basin outlet locations (b, d), respectively.


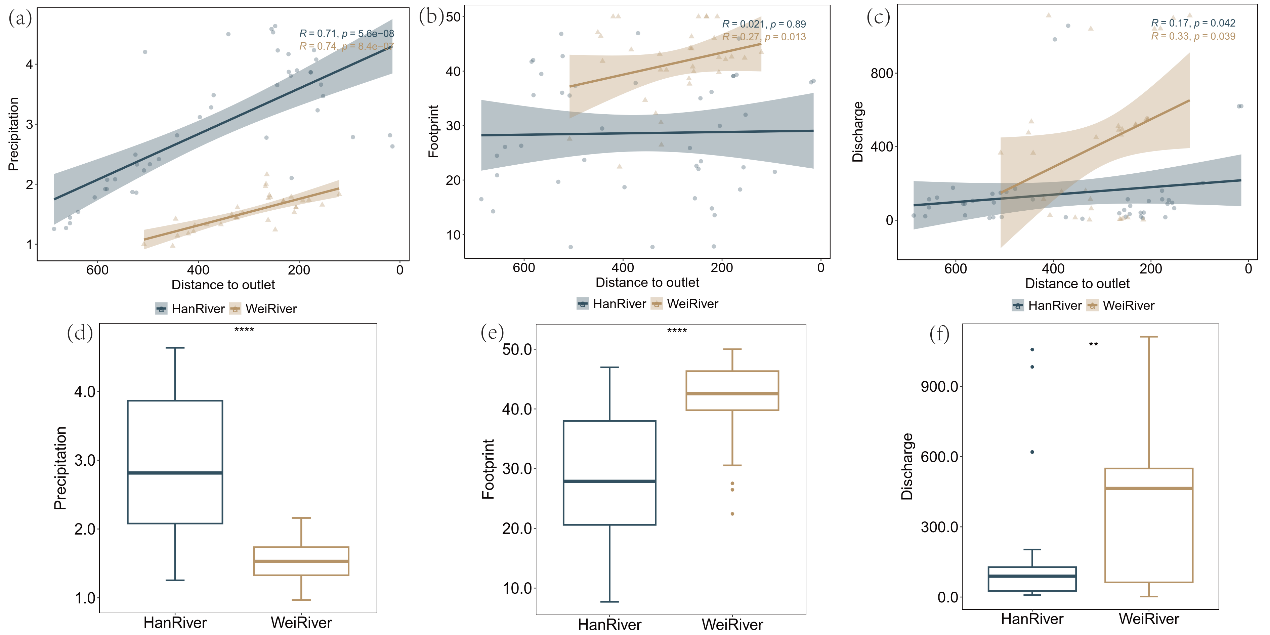


**Figure S3.** The spatial distribution patterns and differences of average precipitation (a, b, c), human footprint (d, e, f) in the Han and Wei River. The spatial distribution patterns represent the average values of average annual precipitation and human footprint within a 1 km buffer zone surrounding each samplesite.


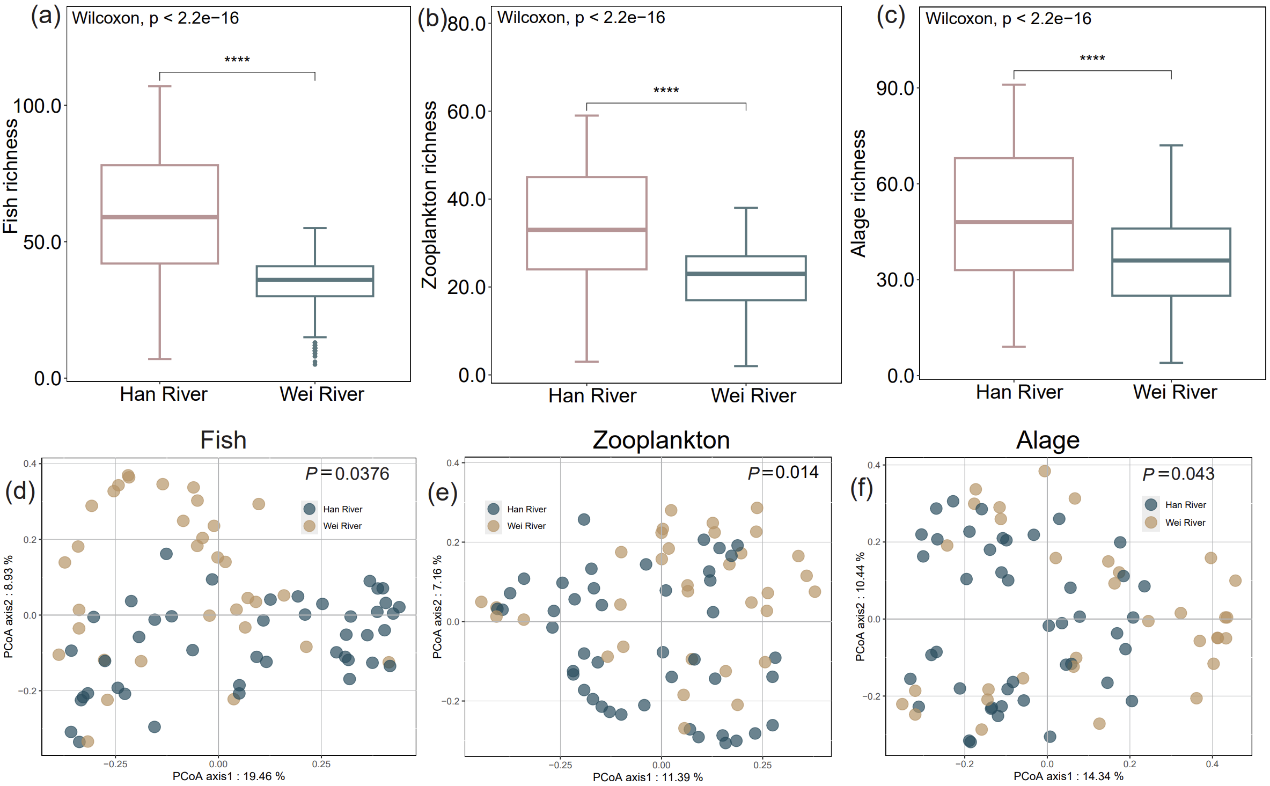
**Figure S4.** The alpha diversity and beta diversity of fish (a, d), zooplankton (b, e), and eukaryotic algae (c, f) communities between the Han and Wei River.


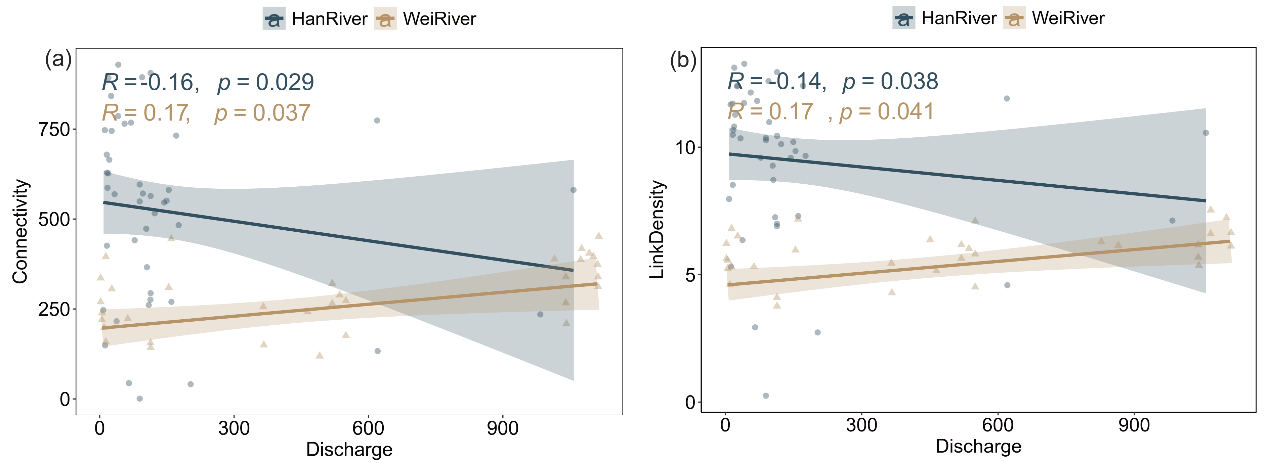


**Figure S5.** Relationship between food web complexity and flow in the Han and Wei River basins (a, b).

**Table S1.** Species names from different trophic levels used in the food web analysis of the Han River Basin.

| Species | Taxa |
| --- | --- |
| *Opsariichthys uncirostris* | Fish |
| *Xenocypris fangi* | Fish |
| *Hemiculter leucisculus* | Fish |
| *Gambusia affinis* | Fish |
| *Sarcocheilichthys nigripinnis* | Fish |
| *Onychostoma macrolepis* | Fish |
| *Hemibarbus maculatus* | Fish |
| *Carassius auratus* | Fish |
| *Rhynchocypris lagowskii* | Fish |
| *Misgurnus bipartitus* | Fish |
| *Silurus asotus* | Fish |
| *Pseudobrama simoni* | Fish |
| *Gnathopogon polytaenia* | Fish |
| *Cobitis macrostigma* | Fish |
| *Squalidus argentatus* | Fish |
| *Ctenopharyngodon idella* | Fish |
| *Rhodeus ocellatus* | Fish |
| *Rhinogobius cliffordpopei* | Fish |
| *Zacco platypus* | Fish |
| *Hypophthalmichthys nobilis* | Fish |
| *Cyprinus carpio* | Fish |
| *Triplophysa sellaefer* | Fish |
| *Triplophysa dalaica* | Fish |
| *Pseudobagrus ondon* | Fish |
| *Acheilognathus hypselonotus* | Fish |
| *Coptodon zillii* | Fish |
| *Abbottina rivularis* | Fish |
| *Leptobotia rubrilabris* | Fish |
| *Pseudorasbora parva* | Fish |
| *Misgurnus anguillicaudatus* | Fish |
| *Paracercomonas compacta* | Protozoa |
| *Woronina pythii* | Protozoa |
| *Rhogostoma schuessleri* | Protozoa |
| *Cercomonas sp.* | Protozoa |
| *Korotnevella pelagolacustris* | Protozoa |
| *Vermamoeba vermiformis* | Protozoa |
| *Echinamoeba exundans* | Protozoa |
| *Rhogostoma sp.* | Protozoa |
| *Vannella simplex* | Protozoa |
| *Corbicula fluminea* | Metazoa |
| *Isomermis lairdi* | Metazoa |
| *Adula aff. californiensis MO-2010* | Metazoa |
| *Gibbosula polysticta* | Metazoa |
| *Asplanchna brightwellii* | Metazoa |
| *Brachionus calyciflorus* | Metazoa |
| *Adineta vaga* | Metazoa |
| *Lecane inermis* | Metazoa |
| *Sinantherina socialis* | Metazoa |
| *Sinocalanus sinensis* | Metazoa |
| *Thermocyclops oithonoides* | Metazoa |
| *Austropallene cornigera* | Metazoa |
| *Baetidae sp. Cy2020 sp2* | Metazoa |
| *Navicula arenaria* | Algae |
| *Wislouchiella planctonica* | Algae |
| *Cyclotella striata* | Algae |
| *Fistulifera saprophila* | Algae |
| *Melosira varians* | Algae |
| *Nitzschia palea* | Algae |
| *Discostella nipponica* | Algae |
| *Cladophora sp.* | Algae |
| *Gonium pectorale* | Algae |
| *Fragilaria sp.* | Algae |
| *Navicula sp.* | Algae |
| *Nitzschia linearis* | Algae |
| *Chloromonas subdivisa* | Algae |
| *Stephanocyclus meneghinianus* | Algae |
| *Cellvibrio gandavensis* | Bacteria |
| *Rhodoferax sp. K129* | Bacteria |
| *Fluviicola taffensis* | Bacteria |
| *Acidovorax defluvii* | Bacteria |
| *Acinetobacter sp. IPPW-14* | Bacteria |
| *cyanobacterium LS123* | Bacteria |
| *Flavobacterium terrigena* | Bacteria |
| *Emticicia sediminis* | Bacteria |
| *Comamonadaceae bacterium* | Bacteria |
| *Flavobacterium sp. THG-DN6.19* | Bacteria |
| *Pseudorhodoferax sp.* | Bacteria |

**Table S2.** Species names from different trophic levels used in the food web analysis of the Wei River Basin.

| Species | Taxa |
| --- | --- |
| *Opsariichthys uncirostris* | Fish |
| *Hemiculter leucisculus* | Fish |
| *Gambusia affinis* | Fish |
| *Sarcocheilichthys nigripinnis* | Fish |
| *Onychostoma macrolepis* | Fish |
| *Hemibarbus maculatus* | Fish |
| *Carassius auratus* | Fish |
| *Rhynchocypris lagowskii* | Fish |
| *Misgurnus bipartitus* | Fish |
| *Silurus asotus* | Fish |
| *Gnathopogon polytaenia* | Fish |
| *Cobitis macrostigma* | Fish |
| *Oxyeleotris lineolata* | Fish |
| *Squalidus argentatus* | Fish |
| *Ctenopharyngodon idella* | Fish |
| *Rhodeus ocellatus* | Fish |
| *Rhinogobius cliffordpopei* | Fish |
| *Zacco platypus* | Fish |
| *Hypophthalmichthys nobilis* | Fish |
| *Cyprinus carpio* | Fish |
| *Acheilognathus hypselonotus* | Fish |
| *Coptodon zillii* | Fish |
| *Abbottina rivularis* | Fish |
| *Pseudorasbora parva* | Fish |
| *Misgurnus anguillicaudatus* | Fish |
| *Micropercops swinhonis* | Fish |
| *Rhogostoma schuessleri* | Protozoa |
| *Korotnevella pelagolacustris* | Protozoa |
| *Vermamoeba vermiformis* | Protozoa |
| *Echinamoeba exundans* | Protozoa |
| *Vannella simplex* | Protozoa |
| *Rhogostoma sp.* | Protozoa |
| *Adula aff. californiensis MO-2010* | Metazoa |
| *Gibbosula polysticta* | Metazoa |
| *Lecane inermis* | Metazoa |
| *Asplanchna brightwellii* | Metazoa |
| *Brachionus calyciflorus* | Metazoa |
| *Adineta vaga* | Metazoa |
| *Sinantherina socialis* | Metazoa |
| *Sinocalanus sinensis* | Metazoa |
| *Navicula arenaria* | Algae |
| *Wislouchiella planctonica* | Algae |
| *Cyclotella striata* | Algae |
| *Melosira varians* | Algae |
| *Microglena monadina* | Algae |
| *Gonium pectorale* | Algae |
| *Pandorina morum* | Algae |
| *Nitzschia palea* | Algae |
| *Tetraselmis cordiformis* | Algae |
| *Discostella nipponica* | Algae |
| *Cladophora sp.* | Algae |
| *Navicula sp.* | Algae |
| *Chloromonas subdivisa* | Algae |
| *Stephanocyclus meneghinianus* | Algae |
| *Paludibacter propionicigenes* | Bacteria |
| *Flavobacterium sasangense* | Bacteria |
| *Fluviicola taffensis* | Bacteria |
| *Acidovorax defluvii* | Bacteria |
| *cyanobacterium LS123* | Bacteria |
| *Aeromonas sp.* | Bacteria |
| *Tolumonas auensis* | Bacteria |
| *Flavobacterium sp. THG-DN6.19* | Bacteria |
| *Pseudorhodoferax sp.* | Bacteria |
